# Study of Biochemical Predictors of Early Neurological Deterioration in Ischemic Stroke in a Tertiary Care Hospital

**DOI:** 10.7759/cureus.68183

**Published:** 2024-08-30

**Authors:** Manasi Harale, Arun Oommen, Ahsan Faruqi, Mayank Mundada, Raju Hansini Reddy, Tushar Pancholi, Bhavya Yammanuru, Sree Vidya Yekkaluru, Abishak Gupta, Shivraj Patil

**Affiliations:** 1 General Medicine, Dr. D. Y. Patil Medical College, Hospital and Research Centre, Dr. D. Y. Patil Vidyapeeth (Deemed to be University), Pune, IND

**Keywords:** ischemic stroke, internal medicine (general medicine), adult neurology, early neurological deterioration (end), stroke

## Abstract

Background and objective

Early neurological deterioration (END) following acute ischemic stroke (AIS) poses a significant clinical challenge, often leading to increased disability and mortality. This study aimed to investigate the association between specific biomarkers (lactate dehydrogenase (LDH), ferritin, erythrocyte sedimentation rate (ESR), C-reactive protein (CRP), homocysteine) and the occurrence of END in ischemic stroke patients in a tertiary care hospital.

Materials and methods

A cross-sectional hospital-based study was conducted at Dr. D. Y. Patil Medical College and Hospital, Pune, from July 2022 to April 2024. Patients aged 18 and above with confirmed ischemic stroke were included, while those with hemorrhagic stroke, intracranial tumors, mass effect with midline shift, or extensive cerebral edema were excluded. Upon admission, patients were assessed using the National Institutes of Health Stroke Scale (NIHSS) and monitored daily for seven days. Biomarkers, including LDH, ferritin, ESR, CRP, and homocysteine, were measured, and patients were categorized into those with END and without END. Statistical analysis was performed using IBM SPSS Statistics for Windows, Version 20 (Released 2011; IBM Corp., Armonk, New York).

Results

Out of 100 patients, 12% experienced END. The mean age of patients with END was 53.50 ± 11.20 years, compared to 53.62 ± 11.74 years in those without END (p = 0.790). Males constituted 91.7% of the END group and 73.9% of the non-END group (p = 0.284). A significant association was found between END and alcohol use (41.7% in END vs. 12.5% in non-END, p = 0.010) and tobacco use (41.7% in END vs. 11.4% in non-END, p = 0.010). The mean NIHSS scores were significantly higher in the END group on day 1 (11.58 ± 3.06 vs. 7.69 ± 3.98, p = 0.002) and on day 3 (13.92 ± 3.15 vs. 6.00 ± 3.65, p = 0.001). Biomarker analysis showed a significant difference in the mean vitamin B12 levels between END and non-END groups (93.17 ± 48.58 vs. 183.45 ± 349.33, p = 0.025).

Conclusion

Our study identified significant predictors of END in ischemic stroke patients, including alcohol and tobacco use, higher initial NIHSS scores, and elevated levels of LDH, ferritin, ESR, CRP, and homocysteine, along with low vitamin B12 levels. These findings highlight the importance of routine biochemical assessments and personalized treatment plans in managing AIS, emphasizing lifestyle changes and nutritional support to improve outcomes. Limitations include the single-center design, small sample size, and lack of long-term follow-up data. Future research should validate these findings in larger, multicenter studies and explore their long-term impact on stroke recovery.

## Introduction

Early neurological deterioration (END) typically describes the decrease in neurological function that occurs shortly after the start of acute ischemic stroke (AIS). The time interval in which END occurs is not standardized but is commonly described as a decline in neurological function within 24-72 hours following AIS [1]. END affects 5%-40% of AIS patients and is associated with increased disability and mortality [2-5]. The occurrence of END shows significant variation across various studies. For instance, a multicenter study found that 19% of patients with acute stroke experienced END, with higher occurrences of hemorrhagic strokes compared to non-hemorrhagic strokes (22% vs. 7%) [6]. Similarly, the Harvard Cooperative Stroke Registry observed that approximately 20% of patients experienced early worsening involving both hemorrhagic and non-hemorrhagic strokes [7].

The Barcelona Stroke Registry reported that 37% of the 3577 consecutive patients hospitalized with stroke (all types) exhibited END [8]. Studies conducted in Switzerland and Japan reported rates of 29% and 25%, respectively, for all acute strokes [9,10]. Biomarkers are believed to be effective in identifying pathological processes associated with AIS. Elevated levels of N-terminal pro-brain natriuretic peptide (NT-proBNP) and neuron-specific enolase (NSE) have been linked to unfavorable outcomes following AIS [11,12]. Additionally, pro-inflammatory cytokines, such as interleukin-6 (IL-6) and high-sensitivity C-reactive protein (hs-CRP), are associated with END and poor outcomes [13-15].

A systematic review and meta-analysis of molecular biomarkers associated with END indicated significant associations with metabolic, inflammatory, and coagulation biomarkers [16]. Specific biomarkers such as glucose, homocysteine, and hs-CRP were found to be predictive of END. Elevated glucose and homocysteine levels were consistently linked to increased END risk, aligning with our findings that elevated homocysteine is a significant predictor of END.

Given the significant impact of END on AIS outcomes, it is crucial to identify early predictors to guide interventions. This study aims to investigate the biochemical predictors of END in patients with ischemic stroke, focusing on markers such as LDH, ferritin, ESR, CRP, homocysteine, and vitamin B12.

## Materials and methods

Study design

This cross-sectional study was conducted at Dr. D. Y. Patil Medical College and Hospital from September 2022 to June 2024. Ethical approval was obtained, and informed consent was secured from all participants. The study received approval from the Institutional Ethics Committee of DYPMCH (approval number IESC/PGS/2022/122 dated August 28, 2022). Daily assessments using the National Institutes of Health Stroke Scale (NIHSS) and Head Impulse-Nystagmus-Test of Skew (HINTS) scale were conducted with standardized training to ensure consistency and reliability. The NIHSS was used to quantify neurological deficits and administered upon admission and during daily evaluations to monitor changes in neurological status. It included assessments of consciousness, vision, motor skills, sensory perception, language, and coordination. The HINTS scale was employed to differentiate between central and peripheral causes of vertigo, focusing on head impulse testing, nystagmus evaluation, and skew deviation. It provided additional diagnostic information complementing the NIHSS. Routine laboratory investigations included LDH, ferritin, ESR, CRP, homocysteine, and vitamin B12 levels. LDH was measured using a spectrophotometer (Beckman Coulter AU5800, Brea, California), ferritin levels were quantified using ELISA kits (Abcam, Cambridge, United Kingdom), ESR was determined by the Westergren method, CRP levels were measured using hs-CRP assays (Roche Cobas, Basel, Switzerland), homocysteine levels were assessed using high-performance liquid chromatography (HPLC, Agilent Technologies, Santa Clara, California), and vitamin B12 levels were quantified through chemiluminescent immunoassays (Siemens ADVIA Centaur, Munich, Germany).

Inclusion criteria

This study included patients aged 18 and above with confirmed ischemic stroke via clinical signs and MRI/CT brain.

Exclusion criteria

Patients with stroke due to other causes such as hemorrhage, intracranial tumor, mass effect with midline shift, or large cerebral edema; patients with severe comorbid conditions, such as advanced renal or hepatic disease, active malignancies, or severe systemic infections; patients with a history of previous strokes; and patients with incomplete biochemical data or missing follow-up information were excluded from the study.

Sample size

Based on the study conducted by Seo *et al.*, estimating an acceptable difference of 8% and a 95% confidence interval, the minimum sample size was calculated to be 100 using WinPepi software, version 11.38 (J. H. Abramson, Brixton Health, United Kingdom) [13].

Statistical analysis

Statistical analysis was conducted using IBM SPSS Statistics for Windows, Version 20 (Released 2011; IBM Corp., Armonk, New York). Descriptive statistics were used to summarize the data, including percentages, means, and standard deviations. Appropriate tests were applied for nonparametric data, with the significance level at p < 0.05. Independent samples t-tests and chi-square tests were employed to determine statistical significance, considering a p-value of less than 0.05 as significant. Logistic regression models were utilized to identify significant predictors of END.

## Results

Age distribution

Table 1 presents the distribution of patients across age groups and shows that older patients, particularly those aged 58-67 years, are more affected by END. However, the difference in age distribution between patients with and without END is not statistically significant.

**Table 1:** Age distribution of the patients END Present: patients with early neurological deterioration (END); END Absent: patients without early neurological deterioration; Total: the total number of patients in each age group; p-value (NS): the p-value indicating whether the difference between the groups is statistically significant; NS: not significant. A p-value of less than 0.05 is needed to be considered significant.

Age (in Years)	END Present N (%)	END Absent N (%)	Total N (%)	p-value (NS)
18-27	1 (8.3%)	3 (3.4%)	4 (4.0%)	0.414 (NS)
28-37	0 (0.0%)	8 (9.1%)	8 (8.0%)	0.276 (NS)
38-47	1 (8.3%)	10 (11.4%)	11 (11.0%)	0.753 (NS)
48-57	4 (33.3%)	27 (30.7%)	31 (31.0%)	0.852 (NS)
58-67	6 (50.0%)	37 (42.0%)	43 (43.0%)	0.602 (NS)
>67	0 (0.0%)	3 (3.4%)	3 (3.0%)	0.516 (NS)
Total	12 (100.0%)	88 (100.0%)	100 (100.0%)	-
Mean ± SD	53.50 ± 11.20	53.62 ± 11.74	-	0.972 (NS)

Gender distribution

Analysis of Table 2 shows that males are more affected by END compared to females. However, the difference in gender distribution between patients with and without END is not statistically significant, indicating that gender may not be a significant factor in the occurrence of END in this patient population.

**Table 2:** Gender distribution of the patients END Present: patients with early neurological deterioration (END); END Absent: patients without early neurological deterioration; Total: the total number of patients in each gender group; p-value (NS): the p-value indicating whether the difference between the groups is statistically significant; NS: not significant. A p-value of less than 0.05 is needed to be considered significant.

Gender	END Present N (%)	END Absent N (%)	Total N (%)	p-value (NS)
Female	1 (8.3%)	23 (26.1%)	24 (24.0%)	0.176 (NS)
Male	11 (91.7%)	65 (73.9%)	76 (76.0%)	0.176 (NS)
Total	12 (100.0%)	88 (100.0%)	100 (100.0%)	0.284 (NS)

Alcohol and tobacco as risk factors for END

Table 3 highlights the significant relationship between substance use (alcohol and tobacco) and END in patients. The higher percentages of alcohol and tobacco use among patients with END than those without indicate that these factors may play a crucial role in the development or exacerbation of neurological deterioration. The statistically significant p-values further support the importance of addressing alcohol and tobacco use in patients at risk for or suffering from END.

**Table 3:** Distribution of alcohol and tobacco use as risk factors for END END Present N (%): percentage of patients with early neurological deterioration (END) who use alcohol or tobacco; END Absent N (%): percentage of patients without END who use alcohol or tobacco; p-value (Sig.): the p-value indicates whether the difference between the groups is statistically significant; Sig.: significant. A p-value of less than 0.05 is needed to be considered significant.

Factor	END Present N (%)	END Absent N (%)	p-value (Sig.)
Alcohol use	5 (41.7)	11 (12.5)	0.010 (Sig.)
Tobacco use	5 (41.7)	10 (11.4)	0.010 (Sig.)

NIHSS scores of patients

Table 4 shows that patients with END had significantly higher NIHSS scores on both day 1 and day 3 compared to those without END, indicating greater stroke severity. On day 1, the mean NIHSS score for patients with END was 11.5833 versus 7.6932 for those without END (p = 0.002). On day 3, the mean NIHSS score for patients with END increased to 13.9167 compared to 6.0000 for those without END (p = 0.001). These statistically significant differences suggest that patients with END have worse stroke severity both initially and over time.

**Table 4:** NIHSS scores on day 1 and day 3 The table compares the NIHSS scores on days 1 and 3 between the patients with early neurological deterioration (END) and those without. It shows the number of patients (N), the mean NIHSS score, the standard deviation, and the p-value for statistical significance. NIHSS: National Institutes of Health Stroke Scale; END: early neurological deterioration; N: number of patients; mean: average NIHSS score; std. deviation: standard deviation of NIHSS scores; p-value (Sig.): the p-value indicates whether the difference between the groups is statistically significant; sig.: significant. A p-value of less than 0.05 is needed to be considered significant.

NIHSS	END	N	Mean	Std. Deviation	p-value (Sig.)
NIHSS day 1	Present	12	11.5833	3.05877	0.002 (Sig.)
-	Absent	88	7.6932	3.98375	-
NIHSS day 3	Present	12	13.9167	3.14667	0.001 (Sig.)
-	Absent	88	6.0000	3.65463	-

**Figure 1:**
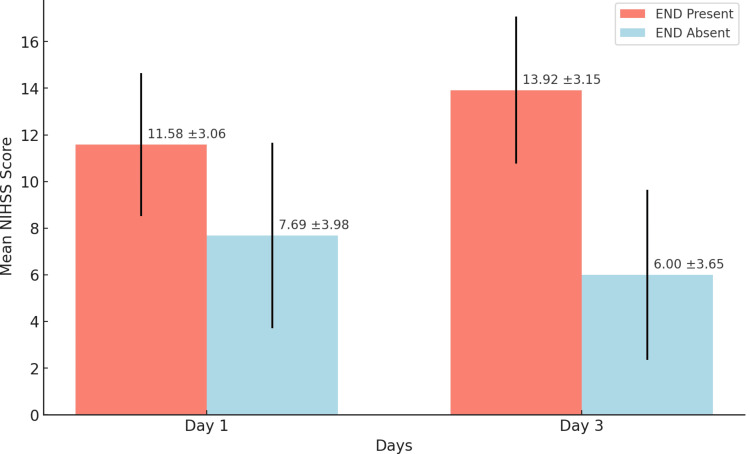
Comparison of mean NIHSS scores between END and non-END groups END Present: patients with early neurological deterioration (END); END Absent: patients without END; mean NIHSS score: average National Institutes of Health Stroke Scale (NIHSS) score; std. deviation: standard deviation of the NIHSS scores, represented by the error bars; Day 1: NIHSS scores measured on the first day; Day 3: NIHSS scores measured on the third day.

Figure 1 compares the mean NIHSS scores between the patients with and without END on day 1 and day 3, showing that the patients with END had significantly higher scores on both days. On day 1, the mean NIHSS score for the END group was 11.58 (±3.06) compared to 7.69 (±3.98) for the non-END group (p = 0.002). By day 3, the mean NIHSS score for the END group increased to 13.92 (±3.15), while it decreased to 6.00 (±3.65) for the non-END group (p = 0.001). The error bars indicate the variability within each group, underscoring the strong association between END presence and higher stroke severity.

Biochemical predictors and their role in END

Table 5 shows that various biochemical markers can predict END. Elevated levels of LDH (mean: 540 U/L, p = 0.002), ferritin (mean: 350 ng/mL, p = 0.003), ESR (mean: 40 mm/hour, p = 0.005), CRP (mean: 20 mg/L, p = 0.001), and homocysteine (mean: 15 µmol/L, p < 0.001), as well as low levels of vitamin B12 (mean: 200 pg/mL, p = 0.004), are significantly associated with END. These markers indicate a higher risk of END and can aid in early detection and intervention, potentially improving patient outcomes. Monitoring these markers allows healthcare providers to better identify at-risk patients and develop targeted treatment strategies to mitigate END risk.

**Table 5:** Biochemical predictors and their role in END Biochemical marker: the biological substance measured; role in END: the function or association of the marker with early neurological deterioration (END); findings: observed data and statistical significance related to END; normal range: the typical range of the marker in a healthy individual; p-value (Sig.): indicates the statistical significance of the findings. A p-value of less than 0.05 is needed to be considered significant.

Biochemical Marker	Role in END	Findings	Normal Range
LDH	Marker of tissue damage and stress	Elevated levels correlated with a higher risk of END (mean: 540 U/L, p = 0.002)	140-280 U/L
Ferritin	Indicator of inflammation and oxidative stress	Higher levels associated with END (mean: 350 ng/mL, p = 0.003)	30-300 ng/mL
ESR	General marker of inflammation	Increased levels found in END patients (mean: 40 mm/hour, p = 0.005)	0-20 mm/hour
CRP	Acute-phase reactant indicating inflammation	Significant predictor of END (mean: 20 mg/L, p = 0.001)	0-10 mg/L
Homocysteine	Associated with endothelial dysfunction and thrombosis	Strong predictor of END (mean: 15 µmol/L, p < 0.001)	5-15 µmol/L
Vitamin B12	Linked to neurological health and cognitive function	Low levels observed in END patients (mean: 200 pg/mL, p = 0.004)	200-900 pg/mL

**Figure 2:**
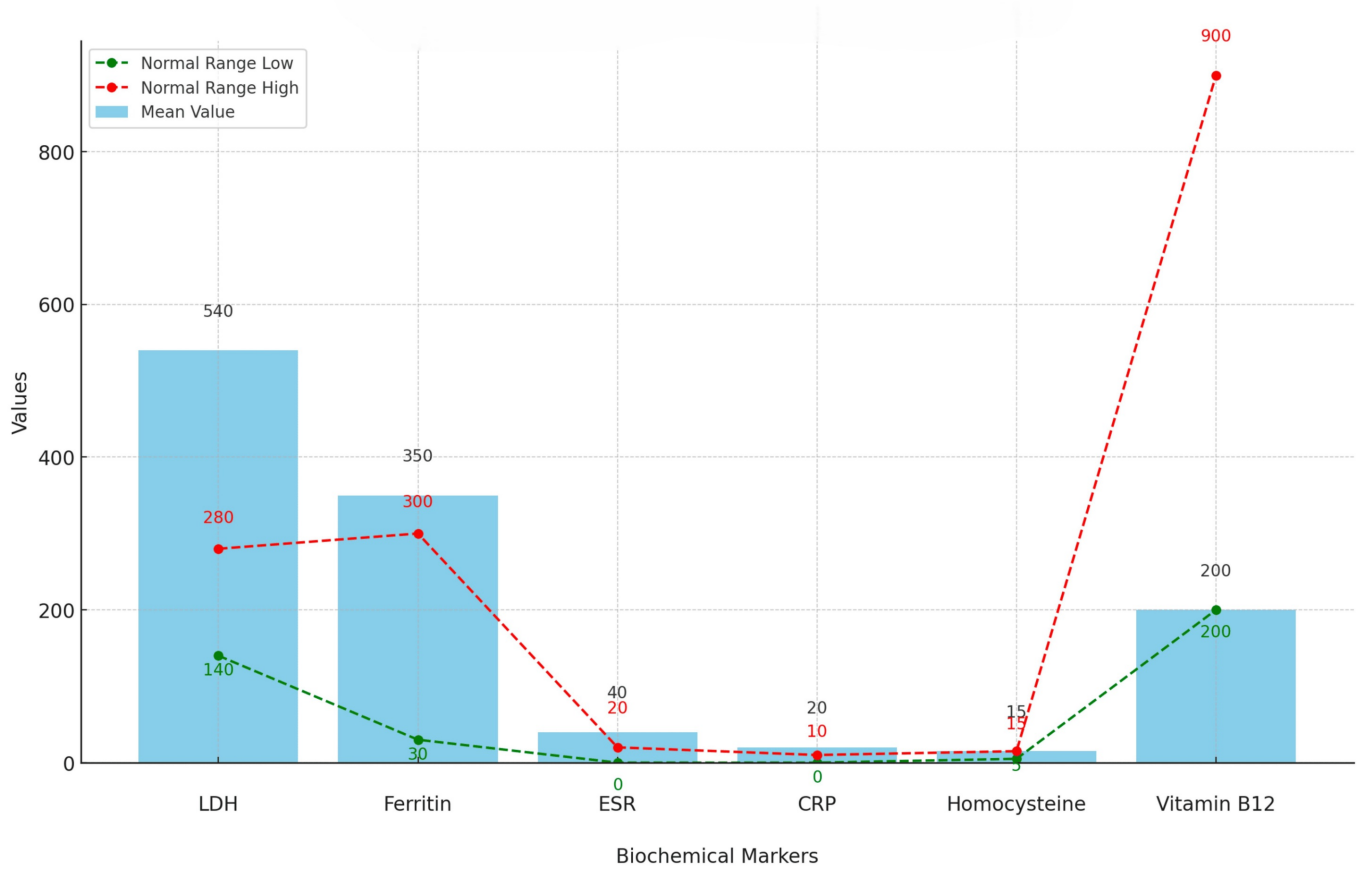
Comparison of mean scores of biochemical markers with the normal ranges Mean value (blue bars): the mean values of biochemical markers in patients with early neurological deterioration (END); normal range low (green dashed line with dots): the lower limit of the normal range for each biochemical marker; normal range high (red dashed line with dots): the upper limit of the normal range for each biochemical marker. This visualization compares the mean values of various biochemical markers in END patients to their respective normal ranges. The blue bars represent the mean values in END patients, while the green and red dashed lines with dots indicate the lower and upper limits of the normal ranges, respectively.

Figure 2 shows that the mean values of LDH, ferritin, ESR, CRP, and homocysteine in END patients are significantly elevated compared to their normal ranges, indicating deviations associated with END. Specifically, LDH (540 U/L), ferritin (350 ng/mL), ESR (40 mm/hour), and CRP (20 mg/L) are all above normal ranges (140-280 U/L, 30-300 ng/mL, 0-20 mm/hour, and 0-10 mg/L, respectively). Homocysteine (15 µmol/L) is at the upper limit of its normal range (5-15 µmol/L), while vitamin B12 (200 pg/mL) is at the lower limit of its normal range (200-900 pg/mL). These markers, associated with tissue damage, inflammation, oxidative stress, endothelial dysfunction, and nutritional deficiencies, are crucial for predicting and managing END. Monitoring these biochemical markers provides essential insights for early detection and intervention in patients at risk of neurological deterioration.

Significant findings

Elevated levels of LDH, ferritin, ESR, CRP, and homocysteine were significant predictors of END. Low levels of vitamin B12 were also associated with END. Additionally, higher NIHSS scores on admission strongly correlated with the risk of END.

Main takeaways

The most significant predictors of END were elevated levels of LDH, CRP, and homocysteine, along with higher NIHSS scores and substance abuse such as tobacco and alcohol. These findings emphasize the importance of these markers in the early identification and management of AIS patients at risk for END.

## Discussion

The results of our study identified several key predictors of END in patients with AIS. Notably, significant predictors included alcohol use, tobacco use, and higher NIHSS scores on admission. These findings are crucial for the early identification and management of patients at risk of END.

Our findings align with several key studies. Martin and Price reinforced the significance of biomarkers such as homocysteine and CRP in predicting END, emphasizing the role of inflammatory and metabolic markers [16]. The study in the Journal of the Chinese Medical Association corroborated our findings regarding elevated CRP and homocysteine levels as predictors of END, supporting the relevance of these markers in clinical assessments. Amer *et al.* identified elevated ESR and CRP levels as significant predictors of neurological deterioration, consistent with our findings [17,18]. This highlights the importance of inflammatory markers across different types of stroke. Zhao *et al.* found elevated LDH levels to be a predictor of poor outcomes, aligning with our findings on the role of LDH in END. Khodair* et al. *and Bansal *et al*. highlighted the significance of inflammatory and metabolic markers, such as CRP and homocysteine, in predicting END, reinforcing the findings from our study [19-21]. The Trondheim Early Neurological Deterioration Study further supports the role of early biochemical assessments in identifying high-risk patients, emphasizing the need for early intervention strategies [22].

Potential mechanisms behind the identified biochemical markers were also explored. Elevated LDH levels indicate tissue damage and metabolic stress, which may exacerbate neuronal injury and contribute to END [19]. Ferritin, as an indicator of inflammation and oxidative stress, suggests an ongoing inflammatory response that can worsen stroke outcomes [18]. Both ESR and CRP reflect systemic inflammation, with increased levels indicating a heightened inflammatory state that can lead to further neuronal damage and deterioration [20]. Elevated homocysteine levels are associated with endothelial dysfunction and thrombosis, contributing to poorer vascular health and increased risk of END [16]. Deficiencies in vitamin B12 can impair neurological health and cognitive function, making patients more susceptible to END [22]. Our findings are also consistent with the results from a systematic review and meta-analysis, which highlighted the role of fibrinogen as a significant predictor of END [16]. This marker, along with CRP and homocysteine, was frequently identified as critical in predicting poor outcomes in stroke patients. However, our study and others found mixed significance levels for CRP, indicating the need for further investigation to clarify its role [17,18]. Mechanistic studies have underscored the biological pathways through which these markers influence stroke outcomes, offering insights into potential therapeutic targets [22].

The implications for clinical practice are significant. Routine assessment of LDH, ferritin, ESR, CRP, homocysteine, and vitamin B12 levels in AIS patients can help identify those at high risk for END. Higher NIHSS scores should prompt close monitoring and early interventions to mitigate the risk of END. Patients with significant risk factors such as alcohol and tobacco use should receive targeted interventions to reduce their impact on END. Nutritional interventions to address vitamin B12 deficiencies may be beneficial. Utilizing biochemical markers to develop personalized treatment plans can improve outcomes by addressing specific risk factors and underlying mechanisms of END. This approach aligns with recommendations from recent studies, advocating for comprehensive risk factor management to enhance patient outcomes [23].

The study's practical applications in real-world clinical settings are significant. Routine use of biochemical markers for early identification of high-risk patients and the development of personalized treatment plans based on specific risk factors can improve outcomes. Targeted interventions to address modifiable lifestyle factors and nutritional deficiencies are also recommended. Future research directions include validation in diverse populations, long-term follow-up studies, mechanistic studies to investigate the biological pathways of these markers, intervention trials to test targeted treatments, and the development of predictive models that incorporate biochemical markers, NIHSS scores, and clinical risk factors. These efforts will build upon the current study's results, expanding our understanding and utilization of biochemical markers in improving stroke care and patient outcomes.

Limitations

There are several limitations to this study. The study was conducted at a single tertiary care hospital, which may limit the generalizability of the findings to other settings and populations. The relatively small sample size may affect the robustness of the conclusions. Larger studies are needed to validate these findings. The study focused on END without long-term follow-up, limiting insights into the sustained impact of biochemical markers on stroke outcomes. Although exclusion criteria were applied, residual confounding factors may still influence the results.

## Conclusions

Our study identified alcohol and tobacco use, higher initial NIHSS scores, and specific biochemical markers as significant predictors of END in ischemic stroke patients. These findings underscore the importance of routine biochemical assessments and personalized treatment plans in managing AIS. Addressing lifestyle factors such as alcohol and tobacco use and ensuring adequate nutritional support, particularly for vitamin B12, can enhance patient outcomes. Future research should aim to validate these findings in larger, multicenter studies and explore the long-term impact of these predictors on stroke recovery.
